# Torsade de pointes during an oral surgery caused by a combination of herbal medicine-induced pseudoaldosteronism and trigeminocardiac reflex

**DOI:** 10.1093/omcr/omad098

**Published:** 2023-09-25

**Authors:** Yohei Kawatani, Kentaro Hoshi, Hitoshi Yamada, Takaki Hori

**Affiliations:** Department of Cardiovascular Surgery, Kamagaya General Hospital, Chiba-Ken, Japan; Department of Dental and Oral-Maxillofacial Surgery, Kamagaya General Hospital, Chiba-Ken, Japan; Department of Anesthesia, Kamagaya General Hospital, Chiba-Ken, Japan; Department of Cardiovascular Surgery, Kamagaya General Hospital, Chiba-Ken, Japan

## Abstract

Licorice can cause pseudoaldosteronism and QT prolongation as its side effect. Trigeminal nerve stimulation, including surgical intervention involving the face, can cause transient bradycardia, known as the trigeminocardiac reflex. Although rare, a combination of these two etiologies can cause ventricular tachycardia. A 50-year-old female patient with a history of hypertension and intake of an herbal drug containing licorice underwent impacted wisdom tooth extraction under general anesthesia. Immediately after placing a month-long prop for visualization in the oral cavity, sinus bradycardia occurred, followed by ventricular tachycardia and torsade de pointes. Mouth prop was removed and cardiopulmonary resuscitation was initiated, and the arrhythmia was resolved. Hypokalemia, metabolic alkalosis and normal aldosterone levels were observed. An inverted T wave and a prominent U-wave were observed on the electrocardiogram. Potassium excretion in urine was normal, although hypokalemia was present. The patient was treated with potassium correction.

## INTRODUCTION

*Glycyrrhiza uralensis* (Licorice in English; Kanzo or 甘草 in Japanese) is a component of some herbal medicines. Using licorice has been reported to cause pseudoaldosteronism, which manifests as hypokalemia and leads to QT prolongation [1]. Furthermore, manipulations during oral surgeries can cause transient bradycardia via the trigeminocardiac reflex (TGR), posing a risk to patients with transient bradycardia and hypotension [2].

Here, we report a case of cardiac arrest caused by a combination of these pathological conditions.

## CASE REPORT

**Figure 1:**
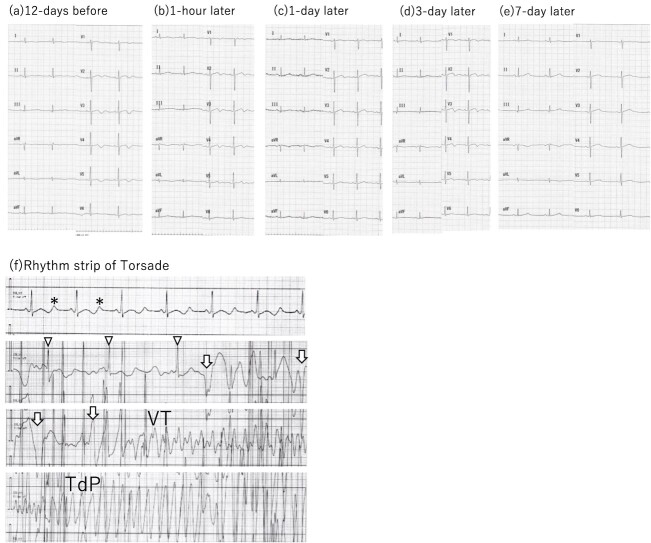
Electrocardiograms. (**a**–**e**) The 12-lead ECG obtained preoperation; 1 h after the operation; 1, 3 and 7 days after the operation. U wave was observed at the preoperative visit. ST-T-U wave morphology became normal along with treatments. (**f**) Rhythm strip at the event. T wave inversion and prominent U wave were observed (*). Sinus bradycardia appeared (arrowhead), followed by premature ventricular beats (arrow mark), leading to ventricular tachycardia (VT) and torsade de pointes (TdP).

A 50-year-old female patient was scheduled for the impacted wisdom tooth extraction. The patient had a history of hypertension, hysterectomy and depression. Losartan potassium (50 mg/day) and eplerenone (50 mg/day) were administered for hypertension. The patient took an herbal drug named ‘Shakuyaku-Kanzoto,’ which contains licorice, for stomach discomfort after the past hysterectomy. The patient reported taking the herbal drug ‘Shakuyaku-kanzoto’ 7.5 g/day (licorice conversion amount; 6.0 mg/day, glycyrrhizin conversion amount 240 mg/day). A preoperative examination performed 12 days before surgery showed mild hypokalemia (3.4 mEq/L; Table 1). Electrocardiogram revealed T wave flattening and U wave (Fig. 1).

**Table 1:** Laboratory examination data

	Preoperation	POD 0	POD 1	POD3	POD7
Blood tests					
Na mEq/L	143	142	139	140	140
K mEq/L	3.4	2.9	3.9	4	4.4
pH	-	7.441	7.421	7.398	-
Renin activity ng/ml/h	-	-	0.2	0.5	2
Aldosterone pg/ml	-	-	11.9	18	15.6
Cortisol μg/dl	-	-	9.13	6.15	9.31
ACTH pg/ml	-	-	3.3	2.8	5.3
Urinary test					
K mEq/L	-	17.4		21.5	29.7

Na, sodium; K, potassium; ACTH, adrenocorticotropic hormone; POD, postoperative day

During surgery under general anesthesia, after setting the mouth prop for visualization of the oral cavity, sinus bradycardia at a heart rate of 40 bpm occurred suddenly, followed by torsade de pointes and ventricular tachycardia (Fig. 1). Mouth prop was removed and cardiopulmonary resuscitation was initiated, and the cardiac rhythm recovered to a normal sinus rhythm shortly. After the surgery, the patient was admitted to the intensive care unit (ICU). Laboratory examination revealed hypokalemia (2.8 mEq/L) and alkalosis (Table 1), and an electrocardiogram showed QT prolongation (Fig. 1).

After ICU admission, the potassium level in urine was not reduced despite the patient having hypokalemia (Table 1), which is characterized by potassium depletion due to elimination through urine. However, the aldosterone level was within the normal range (Table 1).

It was suspected that the sinus bradycardia was caused by vagal nerve stimulation followed by ventricular tachycardia, based on licorice-induced pseudoaldosteronism. Hypokalemia was corrected by administering potassium chloride and magnesium sulfate, with targeted maintenance of over 4.0 mEq/L of potassium. With the correction of hypokalemia, the ST-T-U segment abnormalities disappeared (Fig. 1). The patient did not experience further ventricular arrhythmia after the surgery.

Ischemic heart disease was ruled out using coronary artery-enhanced computed tomography. Ultrasound cardiography and delayed enhanced magnetic resonance imaging of the heart ruled out major myocardial pathologies.

Upon these history and findings, the diagnosis of ventricular arrhythmia caused by a combination of pseudoaldosteronism and TGR was confirmed.

The herbal drug was stopped. Oral potassium agents were continued. Losartan potassium (50 mg/day) and eplerenone (50 mg/day) were restarted after the event. The patient’s condition was fine at the 6-month outpatient follow-up.

## DISCUSSION

Pseudoaldosteronism is an adverse effect of herbal drugs containing licorice. Glycillazine is a pharmacologically active agent in licorice. Licorice intake can result in excess cortisol activity by inhibiting 11β-hydroxysteroid dehydrogenase type 2, an enzyme that inactivates cortisol [1]. The accumulation of activated cortisol subsequently causes the activation of mineral corticoid and glucocorticoid receptors. Finally, potassium excretion and sodium reabsorption are accelerated, causing hypertension, sodium elevation, potassium reduction, metabolic alkalosis and normal aldosterone levels [3]. Subsequently, hypokalemia and QT prolongation are caused by pseudoaldosteronism.

In the present case, mild hypokalemia and hypertension existed before surgery, suggesting that the mineral corticoid effect was elevated. Also, preoperative fasting, cessation of potassium-restoring hypertension treatment agents and extracellular fluid infusion were added before the surgery. Ultimately, clinically significant hypokalemia developed. There is a report of significant hypokalemia and pseudoaldosteronism manifesting during a surgery, even though non-significant pseudoaldosteronism was previously observed in a patient who consumes licorice [4]. We can conclude that there are cases where pseudoaldosteronism becomes significant and disastrous during surgery due to perioperative changes, even though the condition was not significant before surgery. In patients who have consumed licorice before surgery, it is recommended to test potassium levels immediately before surgery.

In the present case, the patient consumed ~6 g/day of licorice for a year. The amount of licorice intake and the development of pseudoaldosteronism have been reported to be correlated. In individuals with a licorice intake of 2, 4 and 6 g/day (80, 160 and 240 mg glycyrrhizin), the incidence of pseudoaldosteronism occurred in 1.7, 3.3 and 11.1% of patients, respectively [5].

Although its incidence rate is low, paroxysmal bradycardia caused by trigeminal nerve stimulation during oral and facial surgery has been reported [2]. Stimulation of any of the sensory divisions of the trigeminal nerve, including setting up the mouth prop [6], is thought to provide a stimulus for the initiation of a sudden cardiac response called TGR, trigeminovagal reflex or oculocardiac reflex. Typically, TGR causes transient bradycardia and hypotension [2]. However, although rare, there are reports of disastrous ventricular arrhythmia being caused by TGR [7]. The treatment for TGR is the removal of trigeminal nerve stimulation. Anticholinergics may also be the treatment of choice. However, anticholinergics cannot provide effective prophylaxis due to their side effects.

QT interval prolongation is a common cause of ventricular arrhythmia. Hypokalemia and bradycardia are well-known causes of QT-interval prolongation. In this case, hypokalemia at the event was at 2.89 mEq/L, which was not severe compared with the previous reports of ventricular arrhythmia caused by pseudo aldosteronism potassium level at 1.4–1.9 mEq/L [8]. In our case, ventricular arrhythmia was assumed to be triggered by TGR based on pseudoaldosteronism advanced by perioperative factors.

In conclusion, hypokalemia can progress perioperatively in patients taking licorice, despite it being mild preoperatively. TGR can trigger transient bradycardia. A combination of these two etiologies can be disastrous. Potassium levels and electrocardiograms should be carefully monitored in such patients during the perioperative period.

Herbal medicines originally have potential risks for side effects [9, 10]. These agents should be administered with care, especially in patients undergoing surgeries.

## References

[1] Ishida T, Kawada K, Morisawa S, Jobu K, Morita Y, Miyamura M. Risk factors for pseudoaldosteronism with Yokukansan use: analysis using the Japanese adverse drug report (JADER) database. Biol Pharm Bull 2020;43:1570–6. 32999167 10.1248/bpb.b20-00424

[2] Shimoda H, Yamauchi K, Takahashi T. Transient asystole associated with vasovagal reflex in an oral surgery patient: a case report. SAGE Open Med Case Rep 2023;11:2050313X221146019. 10.1177/2050313X221146019 PMC9829882 36636097

[3] Hautaniemi EJ, Tahvanainen AM, Koskela JK, Tikkakoski AJ, Kähönen M, Uitto M et al. Voluntary liquorice ingestion increases blood pressure via increased volume load, elevated peripheral arterial resistance, and decreased aortic compliance. Sci Rep 2017;7:10947. 28887501 10.1038/s41598-017-11468-7 PMC5591274

[4] Shibata T, Yoshinuma H, Sugiyama D, Kobayashi O. Severe hypokalemia and metabolic alkalosis caused by licorice discovered during the treatment of intraoperative hypoxemia. Cureus 2022;14:e25432. 35774714 10.7759/cureus.25432 PMC9236722

[5] Mantani N, Oka H, Sahashi Y, Suzuki A, Ayabe M, Suzuki M et al. Relationship between incidence of pseudoaldosteronism and daily dose of Glycyrrhiza. Kampo Med 2015;66:197–202 [Japanese].

[6] Precious DS, Skulsky FG. Cardiac dysrhythmias complicating maxillofacial surgery. Int J Oral Maxillofac Surg 1990;19:279–82. 2124598 10.1016/s0901-5027(05)80420-5

[7] Awasthi D, Roy TM, Byrd RP Jr. Epistaxis and death by the trigeminocardiac reflex: a cautionary report. Fed Pract 2015;32:45–9. 30766072 PMC6363312

[8] Yoshida C, Yamamoto H, Inoue T, Itoh M, Shimane A, Kawai H et al. Torsade de pointes in an older patient with Takotsubo cardiomyopathy caused by licorice-induced pseudoaldosteronism: a case report. Clin Case Rep 2022;10:e6104. 35865783 10.1002/ccr3.6104 PMC9295675

[9] Zakeri MA, Mohammadi V, Bazmandegan G, Zakeri M. Description of ventricular arrhythmia after taking herbal medicines in middle-aged couples. Case Rep Cardiol 2020;2020:6061958–4. 33062339 10.1155/2020/6061958 PMC7547334

[10] Zakeri MA, Bagheripour MH, Iriti M, Dehghan M. Portal vein thrombosis after the consumption of date seed powder: a case study. Case Rep Med 2021;2021:6668722. 33959162 10.1155/2021/6668722 PMC8075688

